# Current and Emerging Treatment Options in Sinus and Nasal Diseases: A Promising Future in the Appropriate Therapies

**DOI:** 10.3390/jcm11247398

**Published:** 2022-12-14

**Authors:** Lin Lin, Lei Cheng

**Affiliations:** 1 Department of Otorhinolaryngology-Head and Neck Surgery, Huashan Hospital of Fudan University, Shanghai 200040, China; 2 Department of Otorhinolaryngology & Clinical Allergy Center, The First Affiliated Hospital, Nanjing Medical University, Nanjing 210029, China

Chronic rhinosinusitis (CRS) is an inflammatory condition of the nose and paranasal sinuses defined by clinical symptoms, including two or more symptoms, one of which should be either nasal blockage or nasal discharge with or without facial pain/pressure or reduction in/loss of sense of smell. Most specialists require both symptoms and auxiliary examinations such as endoscopy and computed tomography to characterize the chronicity as lasting more than 12 weeks [1,2]. The prevalence of CRS is estimated to be 10–28% on the basis of self-reported investigations [3,4] and is approximately 4–9% after the above examinations [5,6,7].

Traditionally, CRS is classified as CRS with nasal polyps (CRSwNP) or without nasal polyps (CRSsNP) based on its phenotype. Now, primary CRS is identified as type 2 or non-type 2 condition according to its endotype [8]. The pivotal factors of type 2 CRS are T helper 2 (Th2) cells and innate lymphoid cells 2 (ILC2s) and their corresponding cytokine productions, including interleukin (IL)-4, IL-5 and IL-13. The increased specific immunoglobulin (Ig) E and eosinophilia in nasal mucosa also characterize the features of the type 2 inflammation [9]. The non-type 2 CRS comprises type 1 and type 3 inflammatory conditions, respectively. The former is mediated by Th1 and its characteristic cytokine interferon gamma (IFN-γ) and ILC1s, and the latter is induced by Th17 and its productions, such as IL-17, IL-22 and IL-23 [10].

With regards to the treatments of CRS, many otorhinolaryngologists suggest performing functional endoscopic sinus surgery (FESS) in order to open up the sinus ostia, drain the purulent discharge and remove nasal polyps after or concomitantly with the pharmacotherapy, such as intranasal corticosteroids (INCS), saline irrigation, antibiotics, allergen immunotherapy and biologics.

A large number of studies support the efficacy of INCS in the treatments of CRSwNP [11,12] and CRSsNP [13]. Agents such as mometasone furoate, budesonide, fluticasone furoate, fluticasone propionate, beclomethasone dipropionate and triamcinolone acetonide can all improve nasal symptoms, and there seem to be no obvious differences among these nasal sprays. INCS should be considered as the first-line therapy for CRS, especially for CRSwNP. As for oral corticosteroids, short courses of this medication can be considered a “medical polypectomy” similar to the surgical procedure in the treatment of CRSwNP [14]. However, a paucity of studies assess the efficacy of oral corticosteroids on nasal symptoms in patients with CRSsNP. Therefore, these agents should be used cautiously in these patients [15].

Nasal saline irrigation is considered to be an adjunctive therapy for CRSwNP or CRSsNP, whether before or after the endoscopic sinus surgery. This medical treatment has been proven to be effective in improving the clinical symptoms, at least through clearing secretions, and inflammatory mediators from the nasal mucosa and promoting mucociliary function in the nasal cavity and paranasal sinuses [16,17]. Relevant experts recommend that high-volume (>200 mL), properly hygienic and thermal, isotonic or hypertonic saline should be used for CRS patients.

Since CRS is not a primary infectious process, antimicrobial therapy is not the mainstream treatment. Some antimicrobials such as macrolide antibiotics and tetracyclines, which are found to have anti-inflammatory properties, may regulate this disease through nonantimicrobial mechanisms. Macrolide antibiotics are reported to have the capability of inhibiting IL-8 and tumor necrosis factor-α productions, downregulating the NF-κB signaling pathway and suppressing neutrophil functions. As a consequence, this agent has been proposed to treat CRSsNP [18,19,20]. However, the current data do not recommend the routine use of the antibiotics in patients with CRSwNP [21].

There is less evidence of efficacy of allergen immunotherapy in the treatment of CRSwNP and CRSsNP. This kind of therapy might not be suggested to be used for the improvement of sinus diseases even if some patients have concomitant allergic rhinitis [22,23].

CRSwNP is most often characterized by elevated local eosinophilia and pronounced infiltrates of Th2 cells and ILC2s [24,25,26] and their type 2 proinflammatory mediators [27], especially in Western countries. Relevant therapies such as biologics targeting type 2 inflammation have been deeply investigated and used to challenge the patients with type 2 CRS, especially eosinophilic CRSwNP.

Many biologics are monoclonal antibodies that are capable of targeting specific inflammatory cells or their mediators involved in the type 2 inflammatory condition [28]. Dupilumab can block IL-4 receptor (R) α, the shared R for both IL-4 and IL-13 signaling pathways, which can accordingly prevent the downstream cascade reactions in the specific immune system cells. This agent was approved by the U.S. Food and Drug Administration (FDA) to treat the difficult-to-control CRSwNP and was also approved to treat moderate-to-severe atopic dermatitis and refractory eosinophilic asthma in June 2019 [29]. Omalizumab is also a popular biologic; it is an anti-IgE monoclonal antibody that binds to free IgE in the blood circulation. It was approved by the FDA for the treatment of CRSwNP, which cannot be adequately controlled using INCS, and for moderate-to-severe persistent allergic asthma and chronic spontaneous urticaria in December 2020 [30]. Mepolizumab is a monoclonal anti-IL-5 antibody that can bind to circulating IL-5, which inhibits the IL-5 signaling pathway and thereby restrains the activation and recruitment of eosinophils. This biological agent was approved by the FDA as an adjunctive therapy for treating patients with CRSwNP in July 2021 [31] and was also approved to be used in the treatment of refractory eosinophilic asthma and EGPA (eosinophilic granulomatosis with polyangiitis) [32]. Benralizumab is a monoclonal anti-IL-5R antibody which was approved by the FDA to treat severe eosinophilic asthma. This agent has also been reported to reduce nasal polyps score, decrease nasal blockage and improve the sense of smell in CRSwNP patients [33]. Since several biologics are available for the therapeutic use for CRSwNP, clinicians must decide which one is appropriate for the individual case. However, no specific biomarkers have been identified to help decide the appropriate treatment using one biological vs. another. Future studies are required to better understand the specific biomarkers, thereby helping guide the clinical therapies and matching the patient’s personalized goals.

There are multiple possible treatment options for patients with CRS. Clinicians should identify which phenotype/endotype the individual patient has in his/her CRS condition based on medical history, endoscopy and computed tomography and a laboratory evaluation. Of course, other factors such as the patient’s preference and the cost and efficacy of the specific therapy should also be taken into consideration before commencing of the most appropriate therapy.

The current Special Issue invites authors to submit their original research including clinical and pre-clinical research papers related to the therapies of sinus and nasal diseases as well as the relevant review manuscripts. The *Journal of Clinical Medicine* also accepts studies showing meaningful but negative results, thereby encouraging scholars and scientists to share these data so that they would not repeat these experiments. We also sincerely hope that all our readers will enjoy this journal.

## Data Availability

Not applicable.
